# A novel vector field analysis for quantitative structure changes after macular epiretinal membrane surgery

**DOI:** 10.1038/s41598-024-58089-5

**Published:** 2024-04-08

**Authors:** Seok Hyun Bae, Sojung Go, Jooyoung Kim, Kyu Hyung Park, Soochahn Lee, Sang Jun Park

**Affiliations:** 1 Department of Ophthalmology, Seoul National University College of Medicine, Seoul National University Bundang Hospital, 173-82 Gumi-ro, Bundang-gu, Seongnam-si, Gyeonggi-do 13620 South Korea; 2 Department of Ophthalmology, HanGil Eye Hospital, Incheon, South Korea; 3 Department of Ophthalmology, Seoul National University College of Medicine, Seoul National University Hospital, Seoul, South Korea; 4 School of Electrical Engineering, Kookmin University, Seoul, South Korea

**Keywords:** Vector field analysis, Epiretinal membrane, Postoperative, Fundus photo, Image processing, Outcomes research

## Abstract

The aim of this study was to introduce novel vector field analysis for the quantitative measurement of retinal displacement after epiretinal membrane (ERM) removal. We developed a novel framework to measure retinal displacement from retinal fundus images as follows: (1) rigid registration of preoperative retinal fundus images in reference to postoperative retinal fundus images, (2) extraction of retinal vessel segmentation masks from these retinal fundus images, (3) non-rigid registration of preoperative vessel masks in reference to postoperative vessel masks, and (4) calculation of the transformation matrix required for non-rigid registration for each pixel. These pixel-wise vector field results were summarized according to predefined 24 sectors after standardization. We applied this framework to 20 patients who underwent ERM removal to obtain their retinal displacement vector fields between retinal fundus images taken preoperatively and at postoperative 1, 4, 10, and 22 months. The mean direction of displacement vectors was in the nasal direction. The mean standardized magnitudes of retinal displacement between preoperative and postoperative 1 month, postoperative 1 and 4, 4 and 10, and 10 and 22 months were 38.6, 14.9, 7.6, and 5.4, respectively. In conclusion, the proposed method provides a computerized, reproducible, and scalable way to analyze structural changes in the retina with a powerful visualization tool. Retinal structural changes were mostly concentrated in the early postoperative period and tended to move nasally.

## Introduction

Epiretinal membrane (ERM) is a thin sheet of fibrous tissue that develops on the surface of the macular area^1^. Some of the membranes contract and cause distortion of the foveal structure and macular edema. The common symptoms include reduced visual acuity, metamorphopsia, and aniseikonia^2^. The standard treatment for ERM is the removal of ERM (membrane peeling) through pars plana vitrectomy to normalize the structure of the macula by eliminating the traction force^3,4^. As retina stretches after ERM removal, displacement of the fovea and retinal vessels is often observed^5,6^.

While accurate understanding of the structural changes, along with the recovery of the distorted retina, might provide new insights into the clinical meaning and prognosis of ERM removal surgery, emerging evidence shows macula displacement and associated changes after ERM removal^7–10^. However, all examinations of retinal images are inevitably inaccurate for comparison between time points or patients due to variations in the degree of magnification or tilting of the image depending on the equipment and the patient's posture.

Moreover, previous studies analyzed the movement of the fovea center only on retinal fundus images or analyzed only a single tomographic image of optical coherence tomography (OCT)^8,10–14^. As ERM not only covers the entire macula area but also varies greatly in direction, extent, and severity from patient to patient, assessing the entire macula is crucial to understanding the structural changes after ERM removal, rather than analyzing a single tomography or fovea center only.

Therefore, we proposed a novel computerized and efficient methodology to assess structural changes in the entire macular region after ERM removal, using two-step registration techniques along with the precise retinal vessel segmentation technique for retina fundus images^15,16^. This novel methodology could provide not only pixel-wise retinal displacement vector field analysis with comprehensive visualization in each patient but also standardized quantification of vector field results for statistical analysis in large-scale and longitudinal studies. Ultimately, the results of this study are expected to enhance the understanding and utilization of macular changes after ERM removal surgery. Additionally, it is expected to contribute to discussions on various clinical applications.

## Materials and methods

### Vector field analysis for quantitative measurement of retinal displacement

Our proposed framework for measuring retinal displacement following ERM removal consists of the following four steps: (1) rigid registration of two retinal fundus images taken before and after ERM removal, (2) Extraction of retinal vessel segmentation from these two retinal fundus images, (3) non-rigid registration of the retinal fundus image taken before surgery in reference to the retinal fundus image taken after surgery, based on the extracted retinal vessel maps. (4) Estimation of the displacement vector field from the retinal fundus image taken before surgery, and calculation of the amount of movement and magnitude of the vector in each pixel of the retinal fundus image taken before surgery.

**Figure 1:**
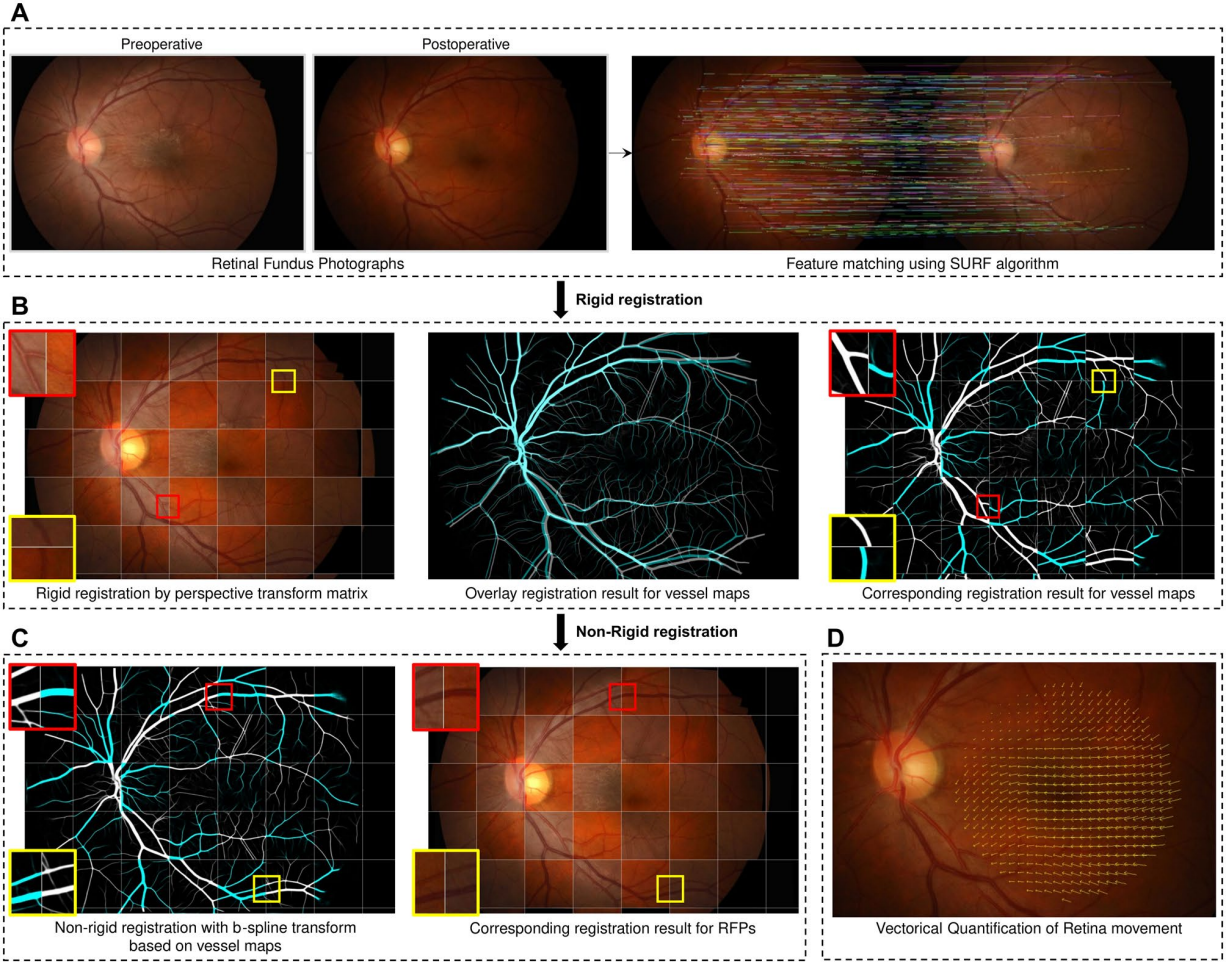
Visual overview for the entire process of proposed vector field analysis. (**A**) Rigid registration of the pair of retinal fundus images was performed by feature point matching using Speeded Up Robust Features. After rigid registration, (**B**) pixel-wise vessel probability maps for retinal fundus images were generated using SSANet through feedforward inference. Given the vessel probability maps, (**C**) non-rigid registration was performed for each pixel in retinal fundus images by applying b-spline transformation model. Finally, (**D**) the pixel-wise amount of retinal movement between two retinal fundus images was calculated.

In all registrations performed with two images, the previous image was matched based on the latest retinal fundus image. An overview of the entire process is shown in Fig. 1.

#### Step 1. Rigid registration of retinal fundus images

Retinal fundus images can be acquired with different angles of view and sizes depending on the types of fundus cameras, viewpoint, and shooting environment, even in the same patient. Theoretically, they can change the angle of view after cataract surgery. Therefore, rigid registration is required to enable analysis in the same environment. The feature point matching problem has been extensively studied, and many methods have been proposed. We used the Speeded Up Robust Features (SURF), which has proven robustness to various image configurations and efficient computational complexity through the comparison of various key point matching in Noh et al^15^. Fig. 1A describes qualitative examples of the feature points detected and matched for the pair of retinal fundus images.

#### Step 2. Retinal vessel segmentation mask extraction from retinal fundus images

After performing projection registration to match the angle of view and size, we apply deformable registration to further refine the pair of retinal fundus images. Since deformable registration would be performed on a retinal vessel map, accurate generation of the retinal vessel segmentation mask is crucial to ensure effective registration. We used the convolutional neural network-based SSANet, trained using the FIREFLY database^17^. The SSANet generated pixel-wise vessel probability map through feedforward inference for retinal fundus images. Figure 1B shows qualitative examples of the vessel probability map generated through SSANet.

#### Step 3. Non-rigid registration of retinal fundus images and displacement vector filed calculation

Using the extracted vessel segmentation masks, we performed non-rigid registration for each pixel in retinal fundus images by applying a b-spline transformation model, with similarity measured as normalized cross-correlation and optimization with the gradient-based L-BFGS-B algorithm (Fig. 1C). We iteratively applied this process in each non-rigid registration task until the vessel segmentation masks of two retinal fundus images were clearly aligned. Next, we can calculate the transformation matrix between the reference frame and the registered frame in the b-spline transform model. Through this transformation matrix, we could obtain the pixel-wise amount of movement between two retinal fundus images **(**Fig. 1D). We named this amount and direction of changes calculated for each pixel of the retinal fundus image as a retinal displacement vector field.

**Figure 2:**
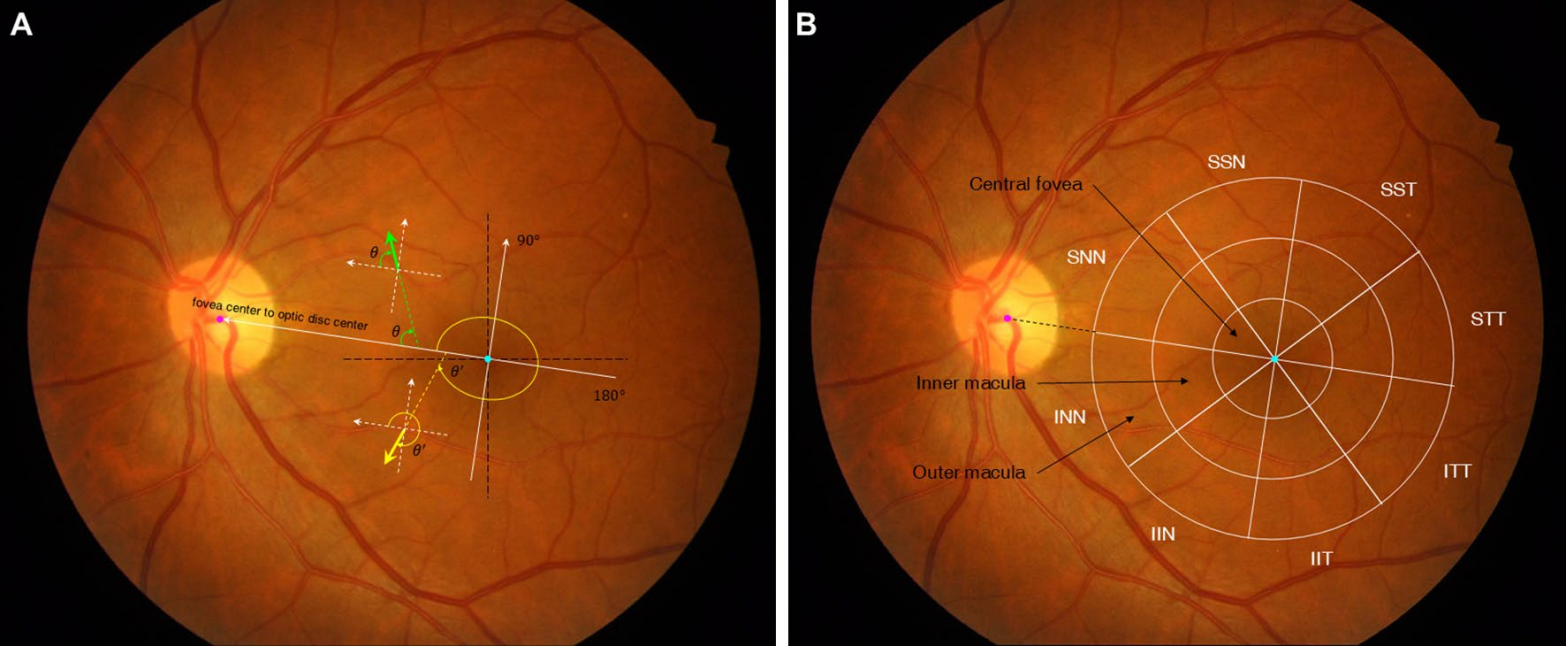
Schematic illustrations of the direction of displacement vectors and the 24 sectors of macula. (**A**) The direction of 0°–360° was defined based on the straight line connecting the fovea center and the optic disc center. Among the pair of retinal fundus images, the vector movement from the first image to the later image was indicated. The nasal side in left eyes was defined as 0°, and the direction it moved was indicated by an angle. Horizontal flipped image was used in the right eye to analyze without distinction between right and left eyes. (**B**) A line connecting the center of optic disc and the fovea was drawn, and concentric circle with a radius of 2/3 of the center of the fovea were defined as the macular region. The radius of the large concentric circle was uniformly divided into thirds and draw two more smaller concentric circles. The innermost concentric circles defined as the central fovea, middle concentric circle as the inner macula, and the outermost as the outer macula. Each concentric circle is divided into 8 directions, and therefore total of 24 sectors was defined.

Since images from both eyes are symmetrical (mirror images), it is necessary to match the image of one eye to the opposite eye for vector calculation. Therefore, a horizontally flipped image was used for the right eye to analyze the data as one. Without this alignment, discrepancies in vector calculations may arise. The direction of the displacement vector indicates the position relative to the disc with respect to the fovea (Fig. 2A). The direction has a value of 0°–360°. In the straight line connecting the center of the fovea and the optic disc, the nasal side was defined as 0°, and the temporal side was defined as 180°. In the direction, 0°–90° means the superonasal direction from the fovea, 90°–180° means the superotemporal direction from the fovea, 180°–270° means the inferotemporal direction from the fovea, and 270°–360° means the inferonasal direction from the fovea.

Finally, in order to accurately evaluate the relative amount of changes for each patient or to deal with images between heterogeneous devices (including ultra-widefield fundus photography), a standardized magnitude was defined. The standardized magnitude is the amount of change when the distance from the fovea center to the disc center of the reference retinal fundus image is set to 1000. The unit of magnitude is in pixels, but as dividing by fovea-to-disc distance (pixels), the unit of standardized magnitude is not separately indicated.

$$Standardized\,Magnitude = \frac{Magnitude \times 1000 \left(pixels\right)}{Fovea-to-disc\,distance (pixels)}$$

#### Step 4. Definition of macular 24 sectors and summarizing displacement vector field

As the current retinal fundus images have a resolution of at least over 1 million pixels, pixel-wise information in the retinal displacement vector field should be summarized in accordance with clinical purposes. Therefore, we defined the region of interest as the macular region, defined as a circle; this circle, where the fovea center is the center of the circle, has a radius of 2/3 of the straight line connecting the center of the fovea and the optic disc (the fovea-to-disc distance). The fovea and optic disc centers were automatically detected using a deep learning-based algorithm^18,19^. When the automatically detected location was not appropriate, the center of the fovea and optic disc was manually adjusted. After that, we divided the macular region (a circle centered on the fovea) into 24 sectors based on 3 concentric circles and 8 directions. Based on the radius of the large concentric circle, it is equally divided into 3 parts and two more smaller concentric circles are drawn. The innermost concentric circle is defined as the central fovea. The middle concentric circle is defined as the inner macula, and the outermost as the outer macula. To compose 24 sectors, 8 directions (supero-naso-nasal (SNN), supero-supero-nasal (SSN), supero-supero-temporal (SST), supero-temporo-temporal (STT), infero-temporo-temporal (ITT), infero-infero-termporal (IIT), infero-infero-nasal (IIN), and infero-naso-nasal (INN)) were defined (Fig. 2B). Then, we calculated the representative vectors of each of the 24 sectors by summing all pixel-wise vectors in each sector. These 24 sectors can be summarized in four regions by grouping 6 sectors each as superior, inferior, nasal, and temporal quadrants or superotemporal, inferotemporal, inferonasal, and superonasal quadrants.

### Study population

The study includes patients with idiopathic ERM who underwent 25-gauge vitrectomy, ERM removal, and internal limiting membrane (ILM) peeling between March 2016 and June 2020. Excluded from the study are patients with secondary ERM, a spherical equivalent exceeding − 6.0 diopters, macular diseases other than idiopathic ERM, other ocular diseases, a history of intraocular surgery unrelated to cataract extraction, presence of postoperative cystoid macular edema, and any other intraocular surgery or intervention conducted within the specified study period.

The study finally involved twenty eyes of twenty patients. They all had ophthalmic examinations before and 1, 4, 10, and 22 months after surgery. The ophthalmic examination included retinal fundus images with a 45° field of view, captured through dilated pupils using the fundus camera VX-10 or VX-10α (Kowa, Co., Ltd., Osaka, Japan). All 20 patients underwent three-port 25-gauge pars plana vitrectomy performed by a retina specialist (KHP) at Seoul National University Bundang Hospital using the Constellation system (Alcon Laboratories, Inc., Fort Worth, TX, USA). After core vitrectomy, ERM and ILM were peeled with intraocular micro-forceps. To minimize the surgical trauma and effects of surgical maneuver, membranes were peeled with a curvilinear fashion. Triamcinolone acetonide (MaQaid, Wakamoto Pharmaceutical Co., Ltd., Tokyo, Japan) was used to enhance visualization of ILM. In all cases, ILM peeling extended farther to the vascular arcades.

### Quantitative evaluation and treating in multiple follow-up images

We should measure the retinal displacement between each time point, as there were four postoperative retinal fundus images (1, 4, 10, and 22 months after surgery) in the present study. To reduce the amount of calculation and obtain intuitive results, we defined the last retinal fundus image taken after surgery as the reference image (those taken 22 months after surgery) and calculated the amount of change between this reference image and the retina fundus images at each time point (those taken before surgery and 1, 4, and 10 months after surgery). After that, we could easily calculate the amount of retinal displacement between other time points by vector operation.

### Statistical analysis

We conducted a statistical analysis using SPSS software, version 26.0. To assess changes in the standardized magnitude at different time points, we employed repeated-measure analysis of variance (ANOVA). Post-hoc analyses, using Wilcoxon signed rank tests, were conducted to compare standardized magnitude across the postoperative periods. A p-value < 0.05 was considered statistically significant.

This study was approved by the institutional review boards of Seoul National University Bundang Hospital (IRB no: B-2205-755-105) and followed the tenets of the Declaration of Helsinki. Informed consent was waived by the institutional review boards of Seoul National University Bundang Hospital due to the retrospective nature of the study.

## Results

**Figure 3:**
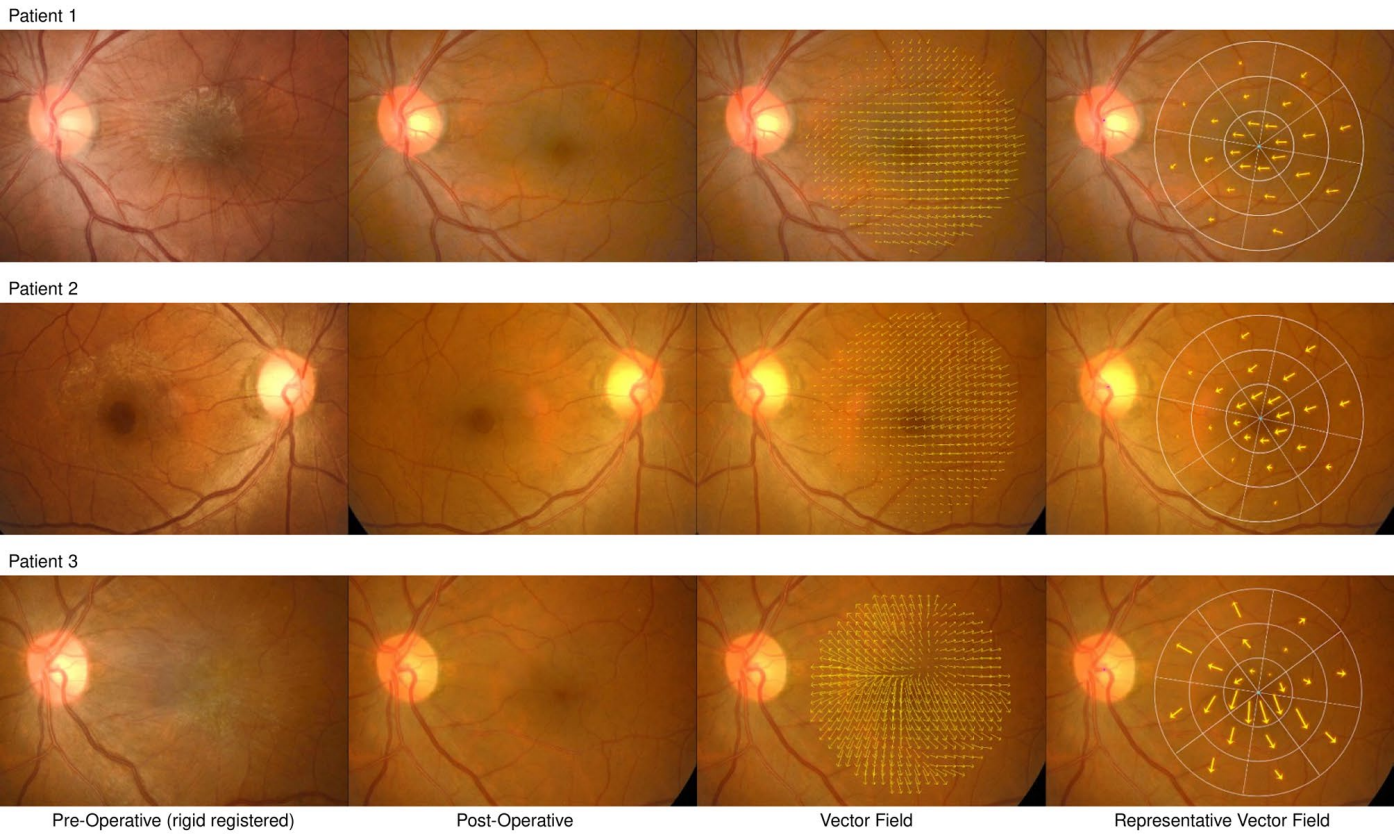
Representative retinal fundus images and visualized displacement vectors after rigid registration. Preoperative (first column) and postoperative 22 months (second column) retinal fundus images after rigid registration in reference to the postoperative images from three patients who underwent epiretinal membrane removal. In the third column, after calculation of pixel-wise retinal displacement of preoperative images in reference to postoperative image, the displacement vectors were visualized in every 25 pixels of presented postoperative retinal fundus images. The summed vectors of each 24 sectors are indicated by arrows in the fourth column. Note that a horizontally flipped image was used for the right eye of patient 2 (second row).

Basic characteristics of the 20 study patients are shown in Supplemental Table S1. The mean age was 63.1 years (range, 46 to 79 years), and 15 patents were women (75.0%). Simultaneous cataract surgery was performed on 3 eyes, and 5 eyes were pseudophakic before the ERM surgery. Figure 3 shows a set of representative retinal fundus images from 3 patients, consisting of preoperative retinal fundus images, postoperative 22-months fundus images, the results of retinal displacement vector field analyses, and those summarized in 24 macular sectors. Video 1 comprises two retinal fundus images taken before and 22 months after surgery, clearly showing that crowed retinal vessels toward the center of ERM in preoperative images are plainly released after ERM removal, even for the large retinal vessels passing over the optic disc head. Additionally, unlike the retinal vessels located on the nasal side of the optic disc that hardly move, the vessels located on the temporal side are crowded before surgery and then released after surgery.

The mean standardized magnitude in the whole macula region from preoperative to postoperative 22 months was 50.6. In the central fovea, inner macula, and outer macula, that was 49.7, 50.7, and 51.4, respectively. The mean direction in the superior 12 sectors ranges from 0.9° to 27.0° and in the inferior 12 sectors from 309.7° to 351.4°, meaning that all sectors move toward superonasal or inferonasal. The mean standardized magnitudes and directions of vectors in each sector are summarized in Supplemental Table S2. The largest displacement sector between preoperative and postoperative 22 months was in SNN of the inner macula (57.7 ± 33.3), and the smallest was in SST of the central fovea (44.7 ± 28.0). Table 1 shows the amount of retinal displacement between time points by secondary vector operation using the values in Supplemental Table S2.

**Table 1:** Mean standardized magnitude and direction of vectors between each time point after secondary calculation (n = 20).

	Preoperative to Postoperative 1mo		Postoperative 1mo to 4mo		Postoperative 4mo to 10mo		Postoperative 10mo to 22mo		P-value*
	Standardized magnitude	direction	Standardized magnitude	direction	Standardized magnitude	direction	Standardized magnitude	direction	
Central fovea	40.1 ± 23.2	347.8	15.1 ± 13.3	352.9	8.5 ± 4.2	341.4	5.1 ± 2.4	335.0	0.000
SST	35.8 ± 23.8	58.5	15.4 ± 14.3	348.0	9.7 ± 6.1	5.7	4.8 ± 3.8	359.4	0.000
STT	39.9 ± 28.9	85.1	21.3 ± 22.3	345.6	12.3 ± 6.7	320.9	5.0 ± 4.8	355.4	0.000
ITT	44.9 ± 30.1	264.4	22.3 ± 25.9	353.7	11.4 ± 5.3	303.1	6.1 ± 4.6	317.5	0.000
IIT	45.4 ± 31.8	280.4	17.9 ± 19.1	359.5	7.6 ± 4.5	331.7	6.0 ± 3.6	314.2	0.000
IIN	39.4 ± 30.6	310.5	12.6 ± 12.0	359.4	6.8 ± 4.4	325.2	5.1 ± 3.8	327.0	0.000
INN	40.5 ± 28.4	332.5	10.9 ± 6.6	352.7	6.5 ± 4.7	338.5	4.8 ± 3.4	331.7	0.000
SNN	38.2 ± 22.6	5.5	9.0 ± 5.0	354.3	5.9 ± 4.0	359.1	4.8 ± 2.2	341.1	0.000
SSN	37.0 ± 25.4	32.9	11.1 ± 9.1	351.3	7.5 ± 5.1	19.4	4.1 ± 3.0	344.3	0.000
Inner macula	38.5 ± 21.8	359.0	14.2 ± 12.4	349.5	7.1 ± 4.0	344.6	5.1 ± 2.6	332.7	0.000
SST	36.9 ± 24.5	50.3	15.5 ± 13.6	339.9	8.8 ± 7.1	341.0	4.9 ± 3.5	337.7	0.000
STT	36.8 ± 22.5	73.5	19.6 ± 18.1	342.8	10.6 ± 8.3	359.8	5.6 ± 4.6	340.9	0.000
ITT	41.0 ± 29.4	276.3	20.0 ± 20.6	347.1	9.4 ± 6.1	330.5	5.6 ± 4.3	319.3	0.000
IIT	42.0 ± 25.7	287.1	15.5 ± 16.4	349.6	6.4 ± 4.2	328.2	5.2 ± 4.0	322.5	0.000
IIN	34.5 ± 27.0	322.5	11.1 ± 11.7	357.8	4.8 ± 3.4	330.7	4.7 ± 3.9	323.7	0.000
INN	41.3 ± 31.5	347.6	10.4 ± 8.1	357.3	5.4 ± 3.0	350.5	5.3 ± 3.7	331.9	0.000
SNN	40.2 ± 29.0	8.9	10.6 ± 7.9	356.0	5.8 ± 2.8	0.6	5.0 ± 2.9	346.0	0.000
SSN	34.9 ± 25.8	29.4	11.4 ± 9.3	348.7	5.9 ± 4.3	344.7	4.6 ± 2.2	337.3	0.000
Outer macula	37.2 ± 20.1	11.6	15.4 ± 13.5	348.5	7.2 ± 5.1	353.9	5.9 ± 3.6	338.0	0.000
SST	38.5 ± 26.5	54.3	16.7 ± 15.0	351.1	8.8 ± 9.1	354.1	6.2 ± 4.3	342.3	0.000
STT	39.3 ± 23.1	58.0	19.2 ± 17.9	344.3	9.8 ± 11.4	3.8	6.2 ± 5.5	343.0	0.000
ITT	37.4 ± 24.8	309.5	18.2 ± 17.7	340.3	9.0 ± 8.9	349.4	6.0 ± 5.2	331.1	0.000
IIT	36.3 ± 17.3	301.2	15.4 ± 15.4	333.9	7.1 ± 4.7	334.4	6.2 ± 4.4	331.8	0.000
IIN	33.9 ± 21.9	327.2	13.1 ± 12.6	342.4	5.5 ± 3.2	344.0	5.6 ± 4.0	327.3	0.000
INN	36.7 ± 24.9	352.5	12.3 ± 11.7	355.3	5.0 ± 3.7	352.1	6.0 ± 3.6	334.5	0.000
SNN	38.8 ± 28.8	17.3	13.0 ± 12.1	0.4	5.8 ± 3.6	358.4	5.2 ± 4.0	349.8	0.000
SSN	36.7 ± 29.9	38.1	14.9 ± 12.7	355.5	6.6 ± 5.4	2.3	5.7 ± 3.7	345.3	0.000

*SST* supero-supero-temporal, *STT* supero-temporo-temporal, *ITT* infero-temporo-temporal, *IIT* infero-infero-termporal, *IIN* infero-infero-nasal, *INN* infero-naso-nasal, *SNN* supero-naso-nasal, *SSN* supero-supero-nasal.

*Repeated-measure ANOVA.

**Figure 4:**
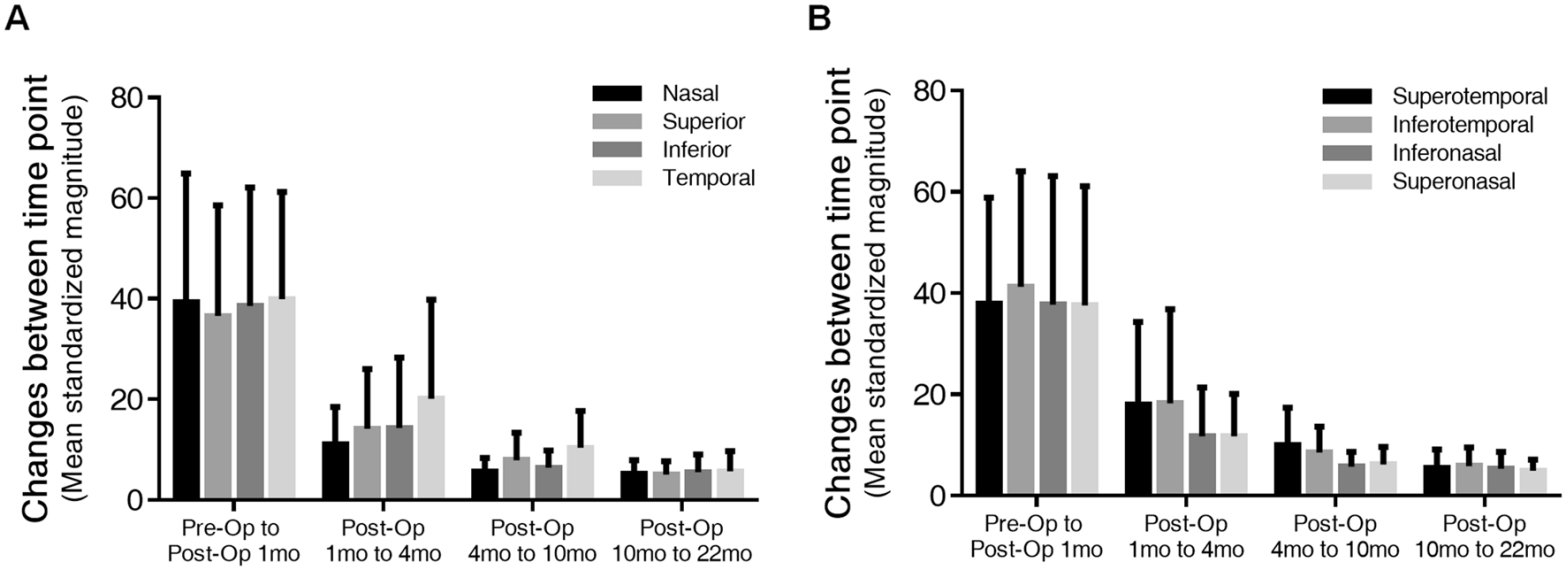
Changes of mean standardized magnitude of displacement vectors between each time points according to four macular quadrants. (**A**) Mean structural changes occurred in superior, inferior, nasal, and temporal quadrants. (**B**) Mean structural changes occurred in superotemporal, inferotemporal, inferonasal, and superonasal quadrants.

The mean standardized magnitude of whole sectors decreased gradually from preoperative to postoperative 1 month (38.6), postoperative 1 month to 4 months (14.9), postoperative 4 months to 10 months (7.6), and postoperative 10 months to 22 months (5.4) (repeated-measure ANOVA, p = 0.000). Except for the postoperative 1 month, which showed a variable amount of direction change, the direction of the vector in all sectors was mostly nasal (superonasal or inferonasal). Displacement was mainly observed in all sectors between preoperative and postoperative 1 month, and the change from postoperative 1 month to 4 months sharply decreased (Wilcoxon signed rank tests, all p < 0.05), with subsequent changes gradually decreasing. Figure 4 shows the amount of displacement between each time points in macular quadrants, and Supplemental Table S3 shows detailed individual values for the standardized magnitudes and directions of the vectors in each quadrant.

## Discussion

The present study introduces a novel vector field analysis methodology for the quantitative and computerized assessment of structure changes after ERM removal. This approach not only provides comprehensive results for the entire macula and its longitudinal changes but also offers informative, intuitive visualization results. The proposed methodology establishes a robust framework for understanding how ERM removal induces retinal structural changes while minimizing the intervention of human experts through the utilization of computer vision and deep learning techniques.

Various methods for measuring retinal displacement after ERM removal have been introduced.^10,13,20,21^ Ichikawa et al.^20^ and Ishida et al.^12^ manually measured distance using near-infrared or fundus autofluorescence images based on the intersection of two retinal vessels. Rodrigues et al. quantified retinal displacement by measuring vertical disc diameter, interarcade distance, fovea-to-disc margin, and perimacular area^21^. Tung et al. used an infrared fundus image to select choroidal markings outside the fovea and then measured foveal movement using a self-designed computer program.^10^ Momota et al. used OCT angiography to match the retinal vasculature around the optic disc to adjust for changes between pre- and post-operative images^8^. Honzawa et al.^22^ utilized a non-rigid registration algorithm constructed with cropped OCT angiography images, employing diffeomorphism. However, these methods may be limited in accuracy due to ongoing changes in retinal vessels, including those passing through the optic disc, after surgery as demonstrated in Video 1. Additionally, there are difficulties in assessing changes in the entire retina, and issues such as image distortion and misalignment seem to be uncorrectable. In contrast, out method quantitatively analyzes the actual movement of each pixel in the entire macular area with minimal human intervention. By automatically marking the disc center and fovea center with a deep learning-based algorithm, out multi-step algorithm calculated the amount of vector shift for each pixel, enabling a more accurate and comprehensive assessment. Rossi et al.^23,24^ applied the Farneback two-frame motion estimation method to consecutive infrared images for en-face displacement calculation. This method may face challenges in cases where the texture features or boundaries in the images are not clear. However, our method, considering the distinct characteristics and patterns of blood vessels, performs non-rigid alignment after initial rigid alignment for a more precise matching.

Gupta et al. revealed that nearly half of patients with ERM showed more than one site of retinal contraction^11^. Kofod et al. divided the infrared retinal fundus image into 25 subfields, recording retinal tangential movement in the nine central subfields corresponding to the macula among the 25 subfields^7^. However, their methods had limitations in assessing longitudinal changes and comparing differences between patients after ERM removal. Measuring vector values from projected retinal fundus images may be limited due to the diversity in individual eye shapes. Our proposed methodology provides measurements of retina displacement as standardized vector values, enabling longitudinal analyses and potential use in investigating natural courses and surgical results of multi-centered ERM. Further studies are warranted.

The present study shows that retinal displacement observed after surgery was most significant during the first month, decreasing significantly over time. This aligns with findings from Lo et al., who reported the greatest macular movement during the first month after surgery in their OCT-based study^13^. Nitta et al. reported that 62.2% of patients with hyperautofluorescent lines, which appeared consistent with the location of the retinal vessels before displacement, vanished within first month after ERM surgery^5^. Our large-scale follow-up study aimed to provide more precise insights into longitudinal changes over time after surgery.

Interestingly, during the first month after surgery, the direction of retinal displacement was diverse. However, thereafter, the direction consistently observed was nasal. This suggests that ERM surgery eliminates ERM-induced centripetal forces and consequent retinal contraction, allowing the retina to stretch within a short period. The changes after ERM removal can be quite different in each sector depending on the location of the ERM center. For instance, in representative Case 3 in Fig. 3, the center of ERM is located inferotemporal to the fovea center, resulting in larger changes (vector magnitude) observed in inferior sectors compared to superior sectors. Although this study analyzed a small number of patients and did not account for the location, number of centers, and severity of ERM, it lays the foundation for future research. The inherent limitation of a small sample size is intentional for this study, aimed at introducing a novel methodology for analyzing retinal displacement after ERM surgery.

Despite these limitations, the study has several strengths. The proposed framework reflects actual macular structural changes after ERM removal throughout the entire macular area. It uses only the fovea and disc centers automatically found in the reference (the last postoperative) image as structural indicators, eliminating the need for researchers to arbitrarily designate other points and reducing the burden on the researcher. The standardized magnitude in the analysis enables direct comparison not only between longitudinal studies in a patient but also between different patients, regardless of the shooting environment and resolution of equipment. The methodology uses standardized vector values, making it easily reproducible with well-known and publicly available techniques. Moreover, it provides a useful tool not only to analyze quantitative changes but also to investigate qualitative characteristics with intuitive visualization.

In conclusion, we propose a computerized approach to quantify and visualize the displacement of retinal structure using retinal fundus images through an efficient framework of vector field analysis consisting of rigid and non-rigid registration techniques and retinal vessel segmentation techniques. The method allows for large-scale cross-sectional and longitudinal studies with minimal computation and expert intervention. The results show that changes in retinal structure after ERM removal concentrate in the early postoperative period, with most of the macular arear tending to move nasally. Our method is expected to contribute to a deeper understanding of ERM and ERM removal surgery in future follow-up studies.

### Supplementary Information

Supplementary Table S1.

Supplementary Table S2.

Supplementary Table S3.

Supplementary Video 1.

Supplementary Legends.

## Data Availability

The data used to support the findings of this study are available from the corresponding author upon request.
