# Supplementary material for: A novel vector field analysis for quantitative structure changes after macular epiretinal membrane surgery
Source: Sci Rep. 2024 Apr 8;14:8242. doi: 10.1038/s41598-024-58089-5 (PMC11002028; doi:10.1038/s41598-024-58089-5)
Supplement: Supplementary file 1 — Supplementary Table S1. [file 41598_2024_58089_MOESM1_ESM.docx]

**Supplemental Table 1** Clinical characteristics of included patients underwent ERM surgery

| Characteristics | Value (n=20) |
| --- | --- |
| Age, mean ± SD | 63.1 ± 11.6 |
| Male:Female | 5:15 |
| Right eyes:left eyes | 11:9 |
| DM (%) | 2 (10.0%) |
| HTN (%) | 7 (35.0%) |
| Previous lens status |  |
| Phakic (eyes, %) | 15 (75.0%) |
| Pseudophakic (eyes, %) | 5 (25.0%) |
| Combined phacovitrectomy (eyes, %) | 3 (20.0%) |
| BCVA (LogMAR), mean ± SD |  |
| Preoperative | 0.18 ± 0.19 |
| Postoperative 1 month | 0.11 ± 0.16 |
| Postoperative 4 months | 0.13 ± 0.10 |
| Postoperative 10 months | 0.09 ± 0.12 |
| Postoperative 22 months | 0.07 ± 0.11 |

ERM = epiretinal membrane; BCVA = best corrected visual acuity; LogMAR = logarithm of the minimum angle of resolution
