# Supplementary material for: A novel vector field analysis for quantitative structure changes after macular epiretinal membrane surgery
Source: Sci Rep. 2024 Apr 8;14:8242. doi: 10.1038/s41598-024-58089-5 (PMC11002028; doi:10.1038/s41598-024-58089-5)
Supplement: Supplementary file 2 — Supplementary Table S2. [file 41598_2024_58089_MOESM2_ESM.docx]

**Supplemental Table 2** Mean standardized magnitude and direction of vectors after registration for all previous frames based on retinal fundus image taken 22 months after surgery (n=20)

|  | Preoperative to postoperative 22mo | | Postoperative 1mo to 22mo | | Postoperative 4mo to 22mo | | Postoperative 10mo to 22mo | |
| --- | --- | --- | --- | --- | --- | --- | --- | --- |
|  | Standardized magnitude | direction | Standardized magnitude | direction | Standardized magnitude | direction | Standardized magnitude | direction |
| Central fovea | 49.7 ± 26.1 | 347.3 | 19.6 ± 11.5 | 346.9 | 10.7 ± 5.9 | 338.0 | 5.1 ± 2.4 | 335.0 |
| SST | 44.7 ± 28.0 | 25.6 | 18.0 ± 11.1 | 352.9 | 10.9 ± 7.3 | 2.0 | 4.8 ± 3.8 | 359.4 |
| STT | 45.3 ± 25.5 | 11.5 | 21.8 ± 16.1 | 344.9 | 12.6 ± 7.6 | 342.6 | 5.0 ± 4.8 | 355.4 |
| ITT | 50.6 ± 31.9 | 316.7 | 24.9 ± 19.8 | 340.8 | 12.2 ± 6.8 | 310.9 | 6.1 ± 4.6 | 317.5 |
| IIT | 55.5 ± 34.8 | 312.1 | 21.1 ± 17.0 | 346.4 | 9.6 ± 7.3 | 321.1 | 6.0 ± 3.6 | 314.2 |
| IIN | 51.5 ± 35.8 | 324.3 | 19.2 ± 14.4 | 342.8 | 10.6 ± 7.6 | 326.1 | 5.1 ± 3.8 | 327.0 |
| INN | 53.4 ± 35.0 | 336.6 | 19.0 ± 10.1 | 343.3 | 10.5 ± 7.1 | 335.4 | 4.8 ± 3.4 | 331.7 |
| SNN | 49.8 ± 28.6 | 0.9 | 16.3 ± 8.0 | 351.9 | 9.5 ± 5.6 | 350.0 | 4.8 ± 2.2 | 341.1 |
| SSN | 47.0 ± 33.9 | 19.9 | 16.4 ± 10.4 | 356.6 | 9.6 ± 6.6 | 2.3 | 4.1 ± 3.0 | 344.3 |
| Inner macula | 50.7 ± 23.3 | 351.9 | 19.2 ± 11.3 | 344.8 | 9.6 ± 5.6 | 338.2 | 5.1 ± 2.6 | 332.7 |
| SST | 47.4 ± 27.1 | 20.4 | 18.9 ± 9.8 | 339.6 | 10.2 ± 7.8 | 339.2 | 4.9 ± 3.5 | 337.7 |
| STT | 45.2 ± 24.4 | 25.6 | 21.4 ± 13.3 | 345.5 | 10.9 ± 8.3 | 350.2 | 5.6 ± 4.6 | 340.9 |
| ITT | 51.8 ± 28.9 | 315.0 | 23.2 ± 16.8 | 338.9 | 10.7 ± 7.1 | 324.6 | 5.6 ± 4.3 | 319.3 |
| IIT | 52.8 ± 29.1 | 309.7 | 19.5 ± 15.8 | 339.7 | 8.9 ± 7.3 | 325.0 | 5.2 ± 4.0 | 322.5 |
| IIN | 46.6 ± 31.3 | 331.2 | 16.2 ± 14.2 | 344.1 | 7.9 ± 7.0 | 326.8 | 4.7 ± 3.9 | 323.7 |
| INN | 56.9 ± 37.7 | 348.1 | 18.6 ± 11.9 | 349.1 | 9.5 ± 6.6 | 340.2 | 5.3 ± 3.7 | 331.9 |
| SNN | 57.7 ± 33.3 | 4.1 | 19.4 ± 10.3 | 354.6 | 9.8 ± 5.5 | 353.0 | 5.0 ± 2.9 | 346.0 |
| SSN | 46.8 ± 29.9 | 13.7 | 16.5 ± 9.7 | 345.1 | 8.7 ± 5.9 | 340.8 | 4.6 ± 2.2 | 337.3 |
| Outer macula | 51.4 ± 27.1 | 359.4 | 20.5 ± 14.7 | 347.3 | 10.3 ± 6.8 | 345.6 | 5.9 ± 3.6 | 338.0 |
| SST | 51.8 ± 33.6 | 27.0 | 20.7 ± 14.2 | 350.1 | 10.3 ± 10.4 | 348.7 | 6.2 ± 4.3 | 342.3 |
| STT | 52.2 ± 28.0 | 23.0 | 22.2 ± 13.4 | 349.1 | 11.7 ± 10.7 | 355.1 | 6.2 ± 5.5 | 343.0 |
| ITT | 49.3 ± 26.8 | 331.0 | 21.4 ± 15.7 | 340.5 | 11.6 ± 9.5 | 340.6 | 6.0 ± 5.2 | 331.1 |
| IIT | 48.2 ± 24.4 | 318.0 | 20.1 ± 16.5 | 333.5 | 10.7 ± 7.1 | 332.9 | 6.2 ± 4.4 | 331.8 |
| IIN | 47.9 ± 30.4 | 332.5 | 19.2 ± 16.2 | 338.9 | 9.0 ± 7.4 | 333.8 | 5.6 ± 4.0 | 327.3 |
| INN | 53.9 ± 34.3 | 351.4 | 19.7 ± 16.4 | 349.6 | 9.6 ± 7.3 | 341.4 | 6.0 ± 3.6 | 334.5 |
| SNN | 56.0 ± 39.6 | 10.2 | 20.5 ± 16.3 | 357.7 | 9.6 ± 6.9 | 353.8 | 5.2 ± 4.0 | 349.8 |
| SSN | 52.1 ± 39.3 | 21.3 | 20.4 ± 15.5 | 355.0 | 9.9 ± 7.7 | 354.2 | 5.7 ± 3.7 | 345.3 |

SST = supero-supero-temporal; STT = supero-temporo-temporal, ITT = infero-temporo-temporal, IIT = infero-infero-termporal; IIN = infero-infero-nasal; INN = infero-naso-nasal; SNN = supero-naso-nasal; SSN = supero-supero-nasal
