# Supplementary material for: A novel vector field analysis for quantitative structure changes after macular epiretinal membrane surgery
Source: Sci Rep. 2024 Apr 8;14:8242. doi: 10.1038/s41598-024-58089-5 (PMC11002028; doi:10.1038/s41598-024-58089-5)
Supplement: Supplementary file 3 — Supplementary Table S3. [file 41598_2024_58089_MOESM3_ESM.docx]

**Supplemental Table 3** Mean standardized magnitude and direction of vectors between each time points for a total of 4 sectors by grouping 6 sectors each (n=20)

|  | Preoperative to Postoperative 1mo | | Postoperative 1mo to 4mo | | Postoperative 4mo to 10mo | | Postoperative 10mo to 22mo | |  |
| --- | --- | --- | --- | --- | --- | --- | --- | --- | --- |
|  | Standardized magnitude | direction | Standardized magnitude | direction | Standardized magnitude | direction | Standardized magnitude | direction | P-value* |
| Subset group 1 |  |  |  |  |  |  |  |  |  |
| Superior | 36.6 ± 22.0 | 42.2 | 14.2 ± 11.8 | 349.3 | 7.9 ± 5.5 | 358.5 | 5.0 ± 2.7 | 5.5 | 0.000 |
| Temporal | 39.9 ± 21.3 | 27.0 | 20.1 ± 19.7 | 346.3 | 10.4 ± 7.3 | 342.0 | 5.7 ± 4.0 | 7.3 | 0.000 |
| Inferior | 38.6 ± 23.5 | 305.9 | 14.3 ± 14.0 | 350.5 | 6.4 ± 3.4 | 331.6 | 5.5 ± 3.5 | 3.4 | 0.000 |
| Nasal | 39.3 ± 25.6 | 358.1 | 11.0 ± 7.5 | 356.3 | 5.7 ± 2.7 | 352.5 | 5.2 ± 2.7 | 2.7 | 0.000 |
| Subset group 2 |  |  |  |  |  |  |  |  |  |
| Superotemporal | 37.9 ± 20.9 | 20.1 | 18.0 ± 16.3 | 13.2 | 10.0 ± 7.4 | 8.8 | 5.4 ± 3.7 | 7.4 | 0.000 |
| Inferotemporal | 41.2 ± 22.8 | 24.0 | 18.2 ± 18.6 | 15.6 | 8.5 ± 5.1 | 6.3 | 5.8 ± 3.7 | 5.1 | 0.000 |
| Inferonasal | 37.7 ± 25.4 | 27.7 | 11.7 ± 9.7 | 9.0 | 5.7 ± 2.9 | 3.4 | 5.3 ± 3.3 | 2.9 | 0.000 |
| Superonasal | 37.6 ± 23.5 | 24.6 | 11.7 ± 8.4 | 8.5 | 6.2 ± 3.4 | 4.2 | 4.9 ± 2.2 | 3.4 | 0.000 |

* Repeated-measure ANOVA
