# Supplementary material for: A novel vector field analysis for quantitative structure changes after macular epiretinal membrane surgery
Source: Sci Rep. 2024 Apr 8;14:8242. doi: 10.1038/s41598-024-58089-5 (PMC11002028; doi:10.1038/s41598-024-58089-5)
Supplement: Supplementary file 5 — Supplementary Legends. [file 41598_2024_58089_MOESM5_ESM.docx]

**Video legend**

Video 1. Serially constructed two rigid-registered retinal fundus images taken before the surgery and 22 months after the surgery. Note that crowded retinal vessels toward the center of ERM are plainly released after ERM removal even for the large retinal vessels passing over optic disc head. (Pre-Op = preoperative; Post-Op = postoperative
